# Beyond Infection—Indoor Airborne Pathogens as Contributors to Respiratory Inflammation and Immune Dysregulation: A Narrative Review

**DOI:** 10.3390/pathogens15080811

**Published:** 2026-08-01

**Authors:** Kalypso-Angeliki Koukouvini, Rafail Fokas, Apostolos Vantarakis

**Affiliations:** Department of Public Health, Medical School, University of Patras, 26504 Patras, Greece; k.koukouvini@gmail.com (K.-A.K.); fokasrafael@gmail.com (R.F.)

**Keywords:** air quality, bioaerosols, airborne transmission, antimicrobial resistance, built environment, exposome, ventilation

## Abstract

Indoor-air research has largely examined infection, microbial ecology, immune effects and antimicrobial resistance separately, leaving the pathway from indoor biological sources to chronic respiratory outcomes insufficiently integrated. This narrative review synthesises evidence on indoor airborne pathogens and non-viable microbial components as health-relevant biological exposures beyond acute infection. Literature published between 2000 and February 2026 was reviewed from PubMed, Scopus and Web of Science, supplemented by guidance from WHO, ECDC, US EPA and ASHRAE. Viable microorganisms and non-viable components, including endotoxin, β-(1→3)-glucans, microbial DNA and extracellular vesicles, engage epithelial pattern-recognition pathways and promote inflammatory signalling. Findings included 6.5% higher TNF-α and 5% higher IL-8 per log-unit increase in fungal-spore exposure among sawmill workers; uncontrolled asthma in 45% of moisture- or mould-exposed versus 33% of non-exposed children; airborne resistance-gene and mobile-element loads of 0.55–479.44 copies/m^3^ in hospital departments; and a 32.8% reduction in viral diversity, but no significant reduction in high viral exposure, following classroom HEPA filtration. These findings support biological plausibility but reveal a fragmented evidence base dominated by observational studies, heterogeneous sampling and limited longitudinal exposure–response data. Indoor bioaerosols should be considered continuous exposures within the exposome, requiring research and regulation across microbiology, environmental engineering, medicine and public health.

## 1. Introduction

People in industrialised societies spend roughly 87 to 90% of their lives inside buildings and another 6 to 7% inside vehicles, leaving only a small proportion of daily time genuinely outdoors [1]. This pattern, first quantified in the National Human Activity Pattern Survey and corroborated in later European and Asian surveys, has reframed the question of environmental health: for most people in most settings, the relevant atmosphere is the indoor one [2]. Yet, for much of the twentieth century, indoor air was treated as a passive backdrop to the “real” environment outside, examined chiefly when something went visibly wrong, such as an outbreak in a hospital, mould in a damp wall or complaints of “sick building syndrome”, rather than as a continuous biological exposure in its own right [3]. The COVID-19 pandemic forced a long overdue reconsideration. The slow, contested recognition that SARS-CoV-2 is transmitted predominantly through inhalation of respiratory aerosols exposed both a scientific blind spot and a regulatory one: building codes, infection control guidance and even basic clinical reasoning had long relied on assumptions about droplet transmission that the underlying physics did not fully support [4,5,6]. The pandemic accelerated interest in the broader concept of the indoor bioaerosol, namely the suspension of viruses, bacteria, fungal spores, pollen, cellular debris and the bioactive molecules they carry, as a determinant of public health [7,8]. Although this review focuses on biological agents, airborne microorganisms and microbial fragments behave as aerosol particles, and their suspension, transport, persistence and respiratory deposition are influenced by particle size, relative humidity, temperature and ventilation [9]. Importantly, the health consequences of this bioaerosol extend beyond acute infectious disease. Even when a microorganism cannot replicate because it is dead, fragmented or present at subinfectious doses, its molecular signatures, such as lipopolysaccharide (LPS) from Gram-negative cell walls, β-(1→3)-glucans from fungal hyphae, peptidoglycan, microbial DNA, mycotoxins and extracellular vesicles, interact with airway epithelial cells and resident immune cells through evolutionarily conserved pattern recognition receptors [10,11,12]. The result is a continuous, low-grade dialogue between indoor microbiota and the human respiratory mucosa. This dialogue can be protective, neutral or pathological depending on dose, microbial diversity, host susceptibility and timing across the life course [13,14,15]. A second strand of concern, emerging from microbial ecology and antimicrobial resistance (AMR) research, complicates the picture further. The built environment is not merely a passive container for human microbiota; it is a selective habitat in which surfaces, ventilation systems and cleaning regimes shape the composition of microbial communities and the flux of antimicrobial resistance genes [16,17,18]. Hospitals are the most extreme case, but offices, schools and dwellings are increasingly recognised as reservoirs and conduits for resistant organisms [18,19,20,21]. This narrative review focuses specifically on indoor airborne biological exposures that affect respiratory health through mechanisms extending beyond acute infection. We examine three interconnected domains: (i) viable airborne pathogens and non-viable microbial components, including endotoxins, β-(1→3)-D-glucans, extracellular DNA, mycotoxins and microbial extracellular vesicles; (ii) their interactions with the respiratory epithelium and innate immune pathways; and (iii) their potential contribution to airway inflammation, immune dysregulation and chronic respiratory disease. Evidence concerning pathogen transmission, antimicrobial resistance and engineering controls is included where it directly informs this exposure-to-host-response pathway. The scientific contribution of this review lies in integrating evidence that is commonly examined separately across environmental microbiology, aerosol science, immunology and public health, while distinguishing established associations from mechanistic hypotheses and unresolved research gaps. On this basis, we apply a source–exposure–host response–health outcome framework to the evaluation of indoor bioaerosols within the human exposome.

## 2. Methodology

Literature was identified through structured searches of PubMed, Scopus and Web of Science between January 2000 and February 2026, using combinations of the terms indoor air, bioaerosol, airborne transmission, endotoxin, β-glucan, fungal spore, built environment microbiome, trained immunity, innate immunity, asthma, chronic obstructive pulmonary disease, antimicrobial resistance and ventilation. Where landmark studies pre-dated 2000, notably Riley and Wells’ foundational work on airborne infection [22], they were retained for historical context. We supplemented database searches with hand-screening of the reference lists of major reviews and with grey-literature sources of recognised authority, including WHO guideline documents, ECDC technical reports, ASHRAE position papers, US EPA assessments and selected national agency reports [23,24,25]. Studies were prioritised when they met one or more of the following criteria: (i) provided mechanistic insight into host–bioaerosol interaction, (ii) reported population-level health outcomes attributable to indoor biological exposure, (iii) evaluated environmental or technological mitigation strategies, or (iv) addressed health equity, vulnerable populations or antimicrobial resistance. We aimed for representation across geographic regions, climates and building typologies, while acknowledging that the evidence base remains skewed toward high-income, temperate-climate settings, a limitation that is revisited in Section 10. As this is a narrative review, no primary experimental, clinical or epidemiological data were generated. No human participants, animals or interventionary procedures were involved; therefore, ethical approval was not required. No new datasets, computer code or laboratory protocols were produced as part of this work. To maintain a focused scope, we included studies addressing airborne or resuspendable biological agents in occupied indoor environments and their associations with respiratory epithelial, inflammatory, immunological, infectious or chronic respiratory outcomes. Studies focusing exclusively on outdoor air pollution, chemical pollutants, thermal comfort, general building performance or non-airborne transmission were excluded, unless these factors directly modified the generation, persistence, deposition or biological effects of indoor bioaerosols. Engineering, surveillance and mitigation studies were retained only when they provided evidence relevant to biological exposure reduction or respiratory health risk.

## 3. Indoor Airborne Pathogens as Components of the Indoor Exposome

The concept of the exposome, introduced by Wild in 2005 and refined since, was developed to complement genomics by capturing the cumulative environmental exposures that shape disease across a lifetime [26,27]. As that concept has matured, it has become clear that the indoor environment dominates much of the exposome in practical terms. A child in a Northern European city, for example, inhales the indoor air of her home, school and car for the great majority of her developing years; her respiratory mucosa is shaped by the microbiota, allergens and combustion by-products of those settings far more than by the outdoor atmosphere alone [28].

### 3.1. Sources and Composition of Indoor Bioaerosols

Indoor air is biologically hybrid. It contains microorganisms shed by occupants, with humans emitting on the order of 10^6^ to 10^8^ bacterial cells per person per hour through breathing, talking, skin shedding and resuspension of settled dust [29,30], together with microorganisms drawn from outdoor air through ventilation, organisms cultivated on damp surfaces, and microorganisms aerosolised from water systems such as cooling towers, showers and humidifiers. These living particles are accompanied by their dead and fragmented counterparts and by an array of bioactive non-viable molecules. Together they form a continuous, dynamic suspension that is sampled by every breath an occupant takes.

### 3.2. Exposure Characteristics and Exposome Implications

Several characteristics distinguish indoor bioaerosols from their outdoor analogues. First, their composition is dominated by human-associated and building-associated taxa rather than by soil and plant-derived organisms [31]. Second, residence times are extended: in poorly ventilated rooms, sub-micrometre particles can remain airborne for hours, allowing repeated re-inhalation and slow dispersion through interconnected spaces [9]. Third, concentrations are highly heterogeneous, with strong gradients near occupants; contaminated surfaces and ventilation supply diffusers [32]. This spatial variability was further illustrated by a culture-based assessment spanning six university microenvironments and 72 indoor and outdoor samples, in which mean indoor bacterial concentrations ranged from 924 CFU/m^3^ in the library to 2750 CFU/m^3^ in the canteen, whereas mean indoor fungal concentrations ranged from 656 to 1799 CFU/m^3^ [33]. Fourth, the relationship between bioaerosol concentration and health risk is non-linear and depends on particle size, viability, biologically active fragments and host susceptibility [12,34]. Conceptualising indoor airborne pathogens as part of the exposome has at least three practical consequences. First, it encourages epidemiological designs that integrate environmental sampling with biomarkers of inflammation and immune activation. Second, it supports a shift from single-pollutant regulation toward composite indices of indoor environmental quality. Finally, it places the design and operation of buildings, historically a matter of energy efficiency, comfort and aesthetics, squarely within public health.

## 4. Major Biological Agents in Indoor Air

A working taxonomy of indoor airborne biological agents is essential before discussing health effects, because dose–response relationships, sampling methods and mitigation strategies differ markedly between groups [12,31]. Although chemical and physical indoor air stressors, including particulate matter, radon, volatile organic compounds, humidity and ventilation conditions, are important components of the broader indoor exposome, they are not classified here as biological agents. In this review, they are considered only where they modify bioaerosol generation, persistence, deposition, resuspension or host responses.

### 4.1. Respiratory Viruses

Respiratory viruses are the most epidemiologically consequential group, particularly influenza A and B viruses, respiratory syncytial virus (RSV), human rhinoviruses, parainfluenza viruses, adenoviruses, human metapneumovirus and coronaviruses, including SARS-CoV-2, SARS-CoV-1 and MERS-CoV [4,5,35]. These viruses are emitted in respiratory aerosols of widely varying size, from large droplets greater than 100 µm that settle quickly to fine and ultrafine aerosols below 5 µm that remain suspended for prolonged periods. The dominant transmission mode for many respiratory viruses is now understood to include inhalation of the fine aerosol fraction, with classical droplet and fomite routes playing smaller, context-dependent roles [4,5]. Indoor air may also carry enteric viruses, notably norovirus during vomiting events [36], and zoonotic viruses in agricultural or veterinary settings.

### 4.2. Airborne Bacteria

Bacteria reach indoor air primarily through human shedding, resuspension of settled dust and aerosolisation from water reservoirs [29]. The dominant taxa in occupied buildings include skin-associated genera such as Staphylococcus, Corynebacterium and Cutibacterium, and oral–respiratory genera including Streptococcus and Veillonella [30,31]. Hospitals and densely occupied institutional buildings additionally accumulate clinically important taxa such as Acinetobacter baumannii, Pseudomonas aeruginosa, methicillin-resistant Staphylococcus aureus and, in water systems, Legionella pneumophila and atypical mycobacteria [37,38]. Mycobacterium tuberculosis remains a defining example of an indoor airborne pathogen with profound public health consequences [39].

### 4.3. Indoor Fungi and Moisture-Related Exposure

Indoor fungal communities are dominated by Cladosporium, Alternaria, Aspergillus, Penicillium, Wallemia, Aureobasidium and, in damp or water-damaged buildings, Stachybotrys chartarum, Chaetomium and Fusarium [40,41]. Fungi emit spores, hyphal fragments and secondary metabolites, including mycotoxins such as satratoxins, ochratoxins and trichothecenes [42]. Outdoor concentrations of Cladosporium and Alternaria are seasonally high and infiltrate buildings through ventilation, while Aspergillus and Penicillium are more characteristically indoor-associated [43]. Visible mould growth on walls, ceilings, window frames or bathroom surfaces may indicate moisture-related microbial amplification indoors and has been associated with respiratory symptoms, respiratory infections and asthma exacerbation in susceptible individuals [42]. Recent field measurements across four Malaysian day-care centres illustrate the magnitude and distinct determinants of these exposures: the highest recorded indoor concentrations reached 1668 CFU/m^3^ for bacteria and 1706 CFU/m^3^ for fungi, while bacterial concentrations were positively correlated with occupancy (Spearman’s ρ = 0.55, *p* < 0.01) and fungal concentrations with relative humidity (ρ = 0.71, *p* < 0.01) [44].

### 4.4. Non-Viable Microbial Components and Allergens

A major conceptual advance in indoor bioaerosol science has been the recognition that non-viable microbial material is biologically active. Lipopolysaccharide from Gram-negative bacteria, lipoteichoic acid and peptidoglycan from Gram-positive bacteria, β-(1→3)-D-glucan from fungal cell walls, muramic acid, ergosterol, extracellular DNA and microbial extracellular vesicles all engage host innate immunity even when the parent organisms cannot replicate [11,45,46]. These molecules persist on settled dust, textiles and HVAC components and can be repeatedly resuspended into the breathing zone [12]. Although not pathogens in the strict microbiological sense, house dust mite allergens, cat and dog allergens, cockroach allergens and mouse allergens are quantitatively dominant in many indoor environments and act through allied immunological pathways [47,48]. They are addressed here because their effects are mechanistically intertwined with microbial exposure. Representative quantitative findings on indoor biological exposures and their associated immune and respiratory health effects are summarized in Table 1.

## 5. Mechanisms of Airborne Exposure and Host Interaction

The journey of a pathogen or microbial fragment from emission to host effect involves four broad stages: emission, transport and persistence, deposition in the respiratory tract, and biological interaction with host tissues.

### 5.1. Emission, Transport and Persistence

Humans are dominant sources for occupied buildings; emission rates depend on activity, with speaking, singing, coughing and exercise producing markedly higher aerosol fluxes than quiet breathing [61,62]. Building materials, water systems, dampness and biofilm-laden HVAC components contribute additional emissions, while occupant behaviour, pets, cleaning, cooking and open windows generate further bioaerosol pulses [12,30]. Once emitted, the fate of a bioaerosol particle is governed by its aerodynamic diameter, airflow patterns, temperature and humidity. Particles smaller than approximately 5 µm settle slowly and behave in a gas-like manner on the timescales relevant to indoor transmission, mixing throughout a room and moving with bulk airflow into adjacent zones [9,63]. Larger droplets settle within seconds to minutes, although they can evaporate to smaller droplet nuclei during transport [4,64]. Humidity is critical: many respiratory viruses, including influenza and SARS-CoV-2, remain infectious longest at low relative humidity, partly because evaporation alters solute concentration and pH within droplets in ways that affect viral stability [65,66]. Recent full-scale chamber experiments further indicate that this relationship is strongly humidity- and time-dependent. In a 29 m^3^ chamber using aerosolised Phi6 as an enveloped SARS-CoV-2 surrogate, infectivity at 25% relative humidity was at least 1 log_10_ higher than at all other tested humidity levels; increasing relative humidity from 25% to 45% reduced infectivity by 2–3 log_10_, while no infectious virus was recovered after 1.5–2 h at 75–85% relative humidity [67]. Temperature, ultraviolet radiation, ozone and surface interactions further modulate persistence.

### 5.2. Respiratory Deposition

Where an inhaled particle deposits in the respiratory tract strongly influences its biological effect. Particles larger than 10 µm deposit predominantly in the nasopharynx; particles between 2.5 and 10 µm reach the tracheobronchial region; and particles smaller than 2.5 µm, including many viral aerosols and microbial fragments, can reach the alveoli [68]. This stratification matters clinically: tuberculosis transmission depends on droplet nuclei reaching the alveoli; allergic rhinitis and acute viral upper-respiratory illness reflect upper-airway deposition; and asthma exacerbations involve the medium and small airways [12,68].

### 5.3. Epithelial and Immune Interactions

**Figure 1:**
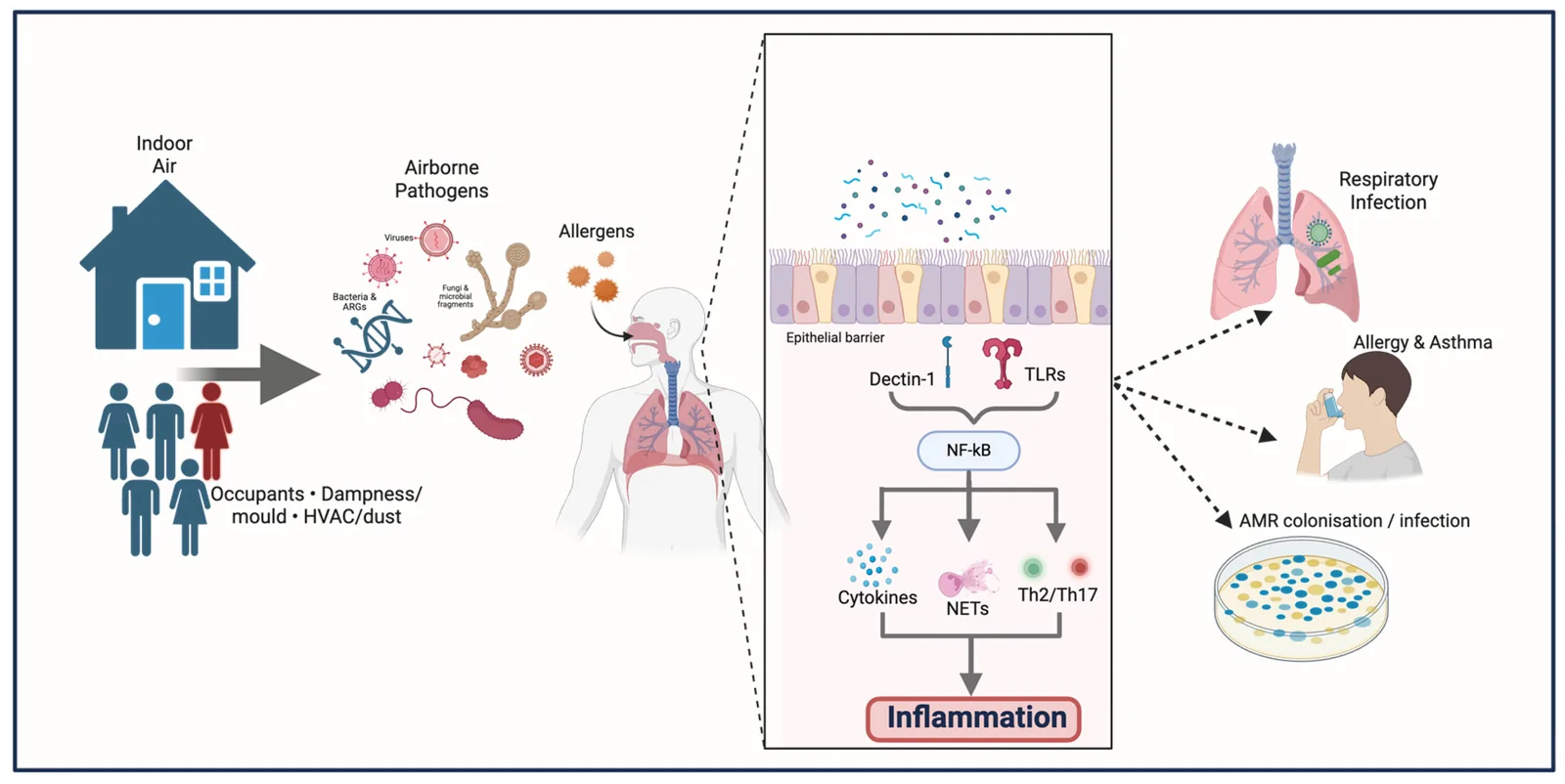
From indoor biological sources to respiratory health outcomes. Airborne pathogens and allergens originating from occupants, dampness and mould, and HVAC-associated dust interact with the airway epithelium and activate Dectin-1-, TLR-, and NF-κB-mediated immune pathways. Cytokine release, NET formation, and Th2/Th17 responses promote inflammation and may contribute to respiratory infection, allergy and asthma, and antimicrobial-resistant colonisation or infection. Created with BioRender.com (https://www.biorender.com/, access on 30 June 2026).

The respiratory epithelium is not a passive surface. Ciliated and goblet cells, club cells, alveolar macrophages and resident dendritic cells form an immunological frontier that recognises microbial molecules through pattern recognition receptors including Toll-like receptors, C-type lectin receptors, NOD-like receptors and RIG-I-like receptors [10,69]. Engagement of these receptors triggers signalling cascades, including NF-κB, MAPK and type I interferon pathways, that orchestrate cytokine release, neutrophil and monocyte recruitment, and antigen presentation to adaptive immunity [11,70]. The mucociliary apparatus, surfactant proteins and antimicrobial peptides provide additional layers of defence [71]. The crucial point for indoor air science is that this defensive system is engaged not only by replicating pathogens but also by microbial fragments and bioactive molecules that cross the airway barrier. Chronic, low-dose stimulation can reshape the basal tone of innate immunity, with consequences that may be protective, neutral or deleterious depending on context. Indoor environments contain diverse biological agents originating from occupants, dampness and mould, HVAC systems, and resuspended dust. Following inhalation, these agents may interact with the airway epithelium and activate innate and adaptive immune pathways, thereby contributing to respiratory inflammation and disease. An overview of these interconnected mechanisms is presented in Figure 1.

## 6. Health Effects of Indoor Airborne Biological Exposure

Indoor airborne biological exposure can affect health through several interconnected pathways, ranging from acute respiratory infection to persistent airway inflammation and longer-term immune dysregulation. The nature and magnitude of these effects depend on the biological agent, exposure intensity and duration, environmental conditions and host susceptibility. This section examines acute respiratory disease, inflammatory and epithelial responses, and emerging evidence concerning long-term immune programming and systemic effects.

### 6.1. Acute Respiratory Infection and Disease

The most direct and best-quantified health consequences of indoor airborne pathogens are acute respiratory infections. The Riley–Wells model, developed from a measles outbreak in a suburban school in the 1970s, remains foundational for thinking about airborne transmission risk and has been refined and extended in the COVID-19 era [22,72]. In modern form, the model expresses the probability of infection as a function of the quantum emission rate of an infectious individual, duration of exposure, breathing rate of susceptible occupants and equivalent ventilation rate of the room, including filtration and germicidal removal [72,73]. Schools, day-care centres, public transport and crowded households are established amplifiers of influenza, RSV, rhinovirus and other seasonal respiratory pathogens [74,75]. The seasonal pattern of influenza is partly explained by indoor crowding during cold weather and by low indoor relative humidity, which can prolong viral viability in air [65,76]. The COVID-19 pandemic provided extensive evidence that indoor air is a major medium of respiratory viral transmission. Documented superspreading events in choirs, restaurants, gyms, meat-processing plants and care homes were typically indoor and associated with poor ventilation, sustained proximity and activities such as singing, loud speaking or exertion that elevate aerosol emission [77,78,79]. The pandemic also catalysed scientific letters and consensus statements from aerosol scientists and engineers urging health authorities to update guidance on airborne transmission and ventilation [6,80]. Mycobacterium tuberculosis remains a canonical airborne pathogen of global public health importance, with millions of new cases annually [39]. Indoor air in prisons, mines, refugee camps and informal urban housing constitutes a major driver of transmission, and ventilation improvements have demonstrable effects on transmission risk in healthcare settings [81]. Legionnaires’ disease, caused by Legionella pneumophila, is acquired through inhalation of aerosols from contaminated water systems, including cooling towers, hot-water systems, decorative fountains and humidifiers [82,83]. Mortality is particularly high in older adults and immunocompromised patients. Hospitals concentrate susceptible hosts and pathogens. Aspergillus is a recognised cause of life-threatening infection in immunocompromised patients exposed to airborne spores during construction or in poorly filtered air-handling systems [84]. Norovirus, measles and varicella have all been documented to spread through airborne or aerosol-associated routes in healthcare settings, and lapses in airborne infection isolation have contributed to SARS-CoV-2 nosocomial outbreaks [85,86]. These examples show that indoor airborne infection is neither a historical concern nor a niche specialty. It is a current, substantial and partially preventable cause of morbidity, mortality and economic loss.

### 6.2. Airway Inflammation, Epithelial Injury and Immune Dysregulation

Even when no overt infection occurs, the airway encounters a continuous stream of microbial material with measurable biological effects. This section synthesises evidence that the inflammatory and immunological consequences of indoor airborne biological exposure constitute an underrecognised public health concern. Endotoxin in settled dust correlates with markers of airway inflammation in occupants of damp buildings, including elevated IL-1β, IL-6, IL-8 and TNF-α in nasal lavage and induced sputum [46,87]. Airborne β-(1→3)-glucans, major structural components of fungal cell walls, are recognised by Dectin-1 (CLEC7A), a C-type lectin pattern-recognition receptor expressed predominantly by macrophages, dendritic cells and neutrophils. Ligand binding activates spleen tyrosine kinase (Syk) and the downstream CARD9–BCL10–MALT1 complex, leading to NF-κB activation, production of inflammatory cytokines such as TNF and IL-6, and the promotion of neutrophil recruitment and Th17 responses [54,57]. In a recent pulmonary exposure model, intranasal β-glucan induced Dectin-1- and CARD9-dependent differentiation of monocyte-derived alveolar macrophages and enhanced their IL-6 response to subsequent stimulation [54]. Dectin-1 activation has also been shown to aggravate neutrophilic airway inflammation in experimental asthma through macrophage pyroptosis and the release of neutrophil-attracting chemokines, accompanied by increased IL-1β, IL-6 and IL-17A expression [55]. These findings provide a mechanistic link between inhaled fungal β-(1→3)-glucans and airway inflammation. However, the magnitude and direction of the response depend on β-glucan structure and accessibility, exposure dose, co-exposures and host susceptibility, while direct evidence from human indoor-exposure studies remains limited [54,55,56,57]. Repeated, sub-clinical engagement of these receptors may maintain a low-grade inflammatory tone that contributes to airway remodelling over time [11,12]. Recent longitudinal occupational evidence supports the possibility of persistent immune modulation at comparatively low exposure levels. In a three-year study involving 450 bioaerosol-exposed and 65 unexposed Norwegian sawmill workers, each log-unit increase in fungal-spore exposure was associated with 6.5% higher serum TNF-α and 5% higher IL-8, while exposed workers also exhibited increased total leukocyte, neutrophil, lymphocyte and basophil counts [52]. Neutrophil extracellular traps (NETs) provide an additional link between innate antimicrobial defence and airway tissue injury. In response to microbial and inflammatory stimuli, activated neutrophils can release decondensed chromatin decorated with histones, myeloperoxidase and neutrophil elastase, forming extracellular scaffolds that immobilise pathogens. Although protective during acute infection, excessive or inadequately cleared NETs expose airway tissues to cytotoxic histones and proteases, disrupt epithelial barrier integrity and amplify cytokine and inflammasome signalling [59,88]. In severe asthma, increased airway NET burden has been associated with epithelial injury, inflammasome activation and IL-1β release [59]. Human rhinovirus-challenge studies and experimental models further indicate that NET formation and the associated release of extracellular double-stranded DNA contribute functionally to airway inflammation, mucus hypersecretion and exacerbation severity in COPD [89]. Recurrent NET formation may therefore sustain a cycle of neutrophil recruitment, tissue injury and chronic airway inflammation, although its specific contribution following long-term indoor bioaerosol exposure remains insufficiently quantified [59,88,89]. Fungal proteases, notably those of Aspergillus and Alternaria, bacterial proteases and viral infections can disrupt epithelial tight junctions, increasing permeability and allowing greater translocation of allergens and microbial molecules [90,91]. This barrier dysfunction is a plausible mechanism linking moisture-damaged buildings to incident asthma in genetically susceptible occupants [17,92]. Repeated viral infection in early life, particularly with rhinovirus and RSV, similarly injures the developing airway and is associated with childhood asthma development [93,94].

### 6.3. Immune Programming, Chronic Inflammation and Emerging Systemic Effects

A particularly important line of evidence concerns the long-term programming of innate immunity by environmental microbial exposure. Children raised on traditional dairy farms, especially Amish children in Indiana, exhibit markedly lower rates of asthma and atopy than genetically related children raised in industrialised dairy operations or in non-farming environments [53]. The protective effect appears to track with exposure to a diverse microbial bioaerosol, including endotoxin, microbial DNA and a wide range of bacterial taxa, and operates through innate immune programming, including macrophage and neutrophil functional reprogramming and changes in haematopoietic progenitors. This phenomenon has been conceptualised as trained immunity: a heritable but non-specific reconfiguration of innate immune cells that primes them for altered responses to subsequent stimuli [95,96]. Indoor microbial exposure, particularly in early life, may represent one route through which innate immune tone and immune tolerance are shaped. Loss of microbial diversity in modern, highly sanitised indoor environments has been hypothesised to contribute to the rising prevalence of allergic and autoimmune disease across industrialised settings [97]. Although epidemiological evidence remains contested, exposure to airborne mycotoxins such as trichothecenes produced by Stachybotrys chartarum has been linked to respiratory and constitutional symptoms in occupants of water-damaged buildings, and in vitro and animal studies confirm cytotoxic and immunomodulatory effects [42,98]. The complex co-exposure pattern in damp buildings, including fungal spores, bacterial endotoxin, β-glucan and volatile organic compounds, makes single-agent attribution difficult and argues for an integrated indoor environmental health perspective. A growing literature links chronic respiratory exposure to inflammatory particles with systemic inflammation, endothelial dysfunction and cardiometabolic risk, paralleling the better-known effects of fine particulate pollution [99,100]. For biological aerosols, the mechanistic plausibility is strong, since systemic spillover of cytokines, translocation of microbial fragments and altered haematopoiesis are all documented consequences of chronic airway inflammation [11,100]. However, this remains an emerging and hypothesis-generating area rather than a fully established causal pathway, and dedicated longitudinal studies are needed. The cumulative implication is that indoor air should be regarded as a continuous immunological exposure with consequences ranging from beneficial training of innate immunity to chronic inflammation and disease. The dose matters, but so do microbial diversity, timing across the life course and host susceptibility.

## 7. Chronic-Disease Exacerbation and Vulnerable Populations

The chronic-disease consequences of indoor bioaerosols are unequally distributed. Several populations are disproportionately affected and merit explicit attention. Asthma is the paradigmatic chronic disease shaped by indoor air. The triad of allergens, fungal exposure in damp homes and viral respiratory infections accounts for many exacerbations and a substantial fraction of incident disease [17,47,101]. Evidence from a comprehensive review supports an association between dampness and visible mould in homes and asthma exacerbation in sensitised children, while the evidence for incident asthma was considered suggestive [17]. Subsequent prospective cohorts have strengthened the case for a causal role of indoor dampness in childhood asthma onset [102,103]. More recently, a multicentre cross-sectional study of 424 children aged 1–17 years with recurrent wheeze or asthma found that 34% had reported home exposure to moisture or mould. Uncontrolled asthma was more frequent among exposed than non-exposed children (45% versus 33%, *p* = 0.03), as were unscheduled healthcare visits during the preceding year (45% versus 32%, *p* = 0.02) [58]. COPD is dominated globally by tobacco smoke and biomass-fuel exposure, but indoor bioaerosols contribute to exacerbations through viral and bacterial respiratory infections [104]. In low-income and middle-income countries, exposure to indoor smoke from solid-fuel combustion is layered onto microbial exposure, with combined effects on airway inflammation and infection risk [105]. Specific occupational exposures, including farmer’s lung, bird fancier’s lung, hot tub lung and metalworking-fluid exposure, exemplify how repeated indoor bioaerosol exposure can drive granulomatous and fibrotic lung disease [106]. Office-building outbreaks of hypersensitivity pneumonitis associated with contaminated humidifiers and ventilation systems are well documented, although now relatively rare [107]. Infants and young children, with developing immune systems and disproportionately high minute ventilation per kilogram of body weight, are particularly susceptible to both the protective and harmful effects of indoor microbial exposure [103,108]. Older adults, especially those with immunosenescence, multimorbidity and reduced mucociliary clearance, are at elevated risk of severe outcomes from indoor airborne infection, as the disproportionate burden of COVID-19 in long-term care facilities demonstrated [86,109]. Immunocompromised patients, including transplant recipients, patients receiving cancer chemotherapy and people with advanced HIV, face elevated risks of opportunistic infection, particularly invasive aspergillosis, from indoor fungal exposure [84]. Indoor air is not equally distributed. Substandard housing, overcrowding, structural dampness, ageing or poorly maintained HVAC systems and the absence of effective filtration are concentrated in low-income communities, ethnic minorities and informal urban settlements [110,111]. The result is that the same populations bearing disproportionate burdens of outdoor air pollution, chronic disease and infectious risk also bear disproportionate indoor biological exposure [111]. Equity considerations are therefore not peripheral to indoor air policy; they are central.

### 7.1. Indoor Air, Microbial Ecology and Antimicrobial Resistance

Buildings are increasingly understood as ecosystems with characteristic microbial communities shaped by occupancy, water, surfaces, ventilation and cleaning [16,112]. In addition, occupants contribute individually distinctive microbial clouds, reinforcing the role of human presence as a major driver of indoor airborne microbial composition [113]. Longitudinal evidence indicates that the microbial signature of a building changes rapidly with the arrival or departure of occupants, with the resident microbiome reorganising the building microbiome around the inhabitants’ skin and oral taxa within hours of moving into a new home [16]. Architectural design influences the diversity and structure of these communities, with mechanically ventilated, isolated spaces showing reduced microbial diversity and increased relative abundance of human-associated taxa compared with naturally ventilated or outdoor-connected spaces [112].

### 7.2. Horizontal Gene Transfer and Antimicrobial Resistance in Indoor Environments

Built environments are also recognised as reservoirs of antimicrobial resistance genes (ARGs). Dust from offices, schools and homes contains a diverse antimicrobial resistance gene repertoire, the abundance of which is correlated with antimicrobial chemicals such as triclosan in cleaning and personal-care products [18,20]. In highly controlled indoor environments, including intensive-care units, increased confinement and cleaning have been associated with reduced microbial diversity and greater diversity of resistance genes, a pattern termed “man-made microbial resistance” [21]. Hospitals are particularly important reservoirs and amplification settings for resistant organisms, including *Klebsiella pneumoniae*, *Acinetobacter baumannii* and other clinically important taxa [114]. Beyond serving as reservoirs, indoor microbial communities may facilitate horizontal gene transfer (HGT) through conjugation, transformation and transduction. Conjugation involves the direct cell-to-cell transfer of DNA, commonly through resistance-bearing plasmids. Recent live-cell imaging demonstrated that biofilm architecture strongly influences the efficiency and spatial dissemination of conjugative plasmids [115]. Transformation enables competent bacteria to acquire extracellular DNA (eDNA) released from lysed or actively secreting cells. Biofilm matrices can retain eDNA and bring potential recipient cells into close proximity, and *Pseudomonas aeruginosa* biofilms have been shown to acquire both chromosomal and plasmid DNA through natural transformation [116]. Integrons are not independently mobile but can capture and express gene cassettes, including ARGs, and are commonly embedded within plasmids or transposons that provide mobility. Experimental evidence indicates that natural transformation can facilitate the interspecies transfer of transposons, class 1 integrons and resistance gene cassettes [117]. Consistent with these mechanisms, a 2025 genomic analysis of 3095 clinical isolates representing more than 100 bacterial species identified resistance-associated mobile genetic elements (MGEs) across multiple taxa and highlighted plasmids and transposons as major vehicles of ARG dissemination [118]. Transduction represents a third HGT route, in which bacteriophages package bacterial DNA and transfer it to recipient cells. However, a 2026 analysis identified ARGs in only 82 of 38,861 phage genomes, indicating that phage-mediated ARG transfer is biologically plausible but that ARG carriage by phages is uncommon and should not be assumed to occur frequently in all environments [60]. Biofilms may therefore function as context-dependent HGT hotspots by concentrating bacterial cells, eDNA and MGEs, although transfer efficiency depends on microbial composition, spatial organisation and environmental selection pressures [115,116,117]. Recent quantitative evidence confirms that indoor air can contain measurable loads of resistance determinants. During an influenza A outbreak, airborne bacterial concentrations across hospital departments ranged from 118 to 259 CFU/m^3^, while the absolute abundance of ARGs and MGEs ranged from 0.55 to 479.44 copies/m^3^ and peaked in respiratory ward air [51]. The most abundant detected determinants included *tetL-02*, *lnuA-01*, *intI1*, *ermB* and *qacEdelta1-02*. Complementing these hospital findings, a 2026 metagenomic study of household airborne dust in four Nordic cities identified persistent macrolide, tetracycline and aminoglycoside resistance genes, city-specific resistome profiles and associations between several ARG classes and outdoor particulate matter [119]. The global health implications are substantial. The 2019 Global Burden of AMR estimated 1.27 million deaths directly attributable to bacterial AMR and 4.95 million associated deaths globally, with respiratory infections among the leading syndromes [120]. Although most AMR transmission is not airborne, indoor air may contribute to AMR dissemination through aerosolisation from indoor reservoirs, dust resuspension and dispersion of resistant skin and respiratory flora [51,119,121]. However, the detection of airborne ARGs or MGEs does not establish that successful HGT occurs during or after airborne transport. The viability of aerosolised bacterial hosts, the persistence and bioavailability of eDNA and the contribution of airborne transport relative to contact, waterborne and clinical transmission routes remain insufficiently quantified. Indoor air management should therefore be considered one component of a broader One Health AMR response [19,122].

## 8. Environmental Determinants of Pathogen Persistence and Exposure

The biological consequences of indoor air cannot be separated from the physical conditions of the spaces in which exposure occurs. Several environmental determinants are critical.

### 8.1. Ventilation and Filtration

Ventilation rate, expressed in air changes per hour or litres per second per occupant, is the single most studied and consequential parameter [123]. Insufficient ventilation prolongs aerosol residence time, elevates carbon dioxide as a tracer of rebreathed air and is associated in epidemiological studies with elevated rates of acute respiratory illness, sick-building symptoms and reduced cognitive performance [124,125]. WHO and ASHRAE have iteratively revised guidance toward higher ventilation rates, particularly after COVID-19 [23,80,126]. However, ventilation by itself is not a panacea; high outdoor air supply in heavily polluted urban environments can introduce particulate matter and outdoor allergens if filtration is inadequate [127]. High-efficiency particulate air filters and high-MERV-rated mechanical filters remove a large fraction of bioaerosol particles and are well tolerated within properly designed HVAC systems [128]. Portable HEPA air cleaners have a strong evidence base for reducing measured airborne particle concentrations and simulated pathogen exposure, and have been evaluated in classrooms and care-home settings [129,130]. However, their effectiveness against real-world viral exposure appears to be context dependent. In a 2025 secondary analysis of a cluster-randomised, placebo-controlled trial involving 200 elementary-school classrooms and 532 bioaerosol samples, respiratory viral targets were detected in 98.5% of samples. HEPA purification was not associated with significantly lower odds of high viral exposure (OR 0.50, 95% CI 0.08–3.25; *p* = 0.46), although it was associated with a 32.8% reduction in viral diversity (β = −1.02, 95% CI −1.68 to −0.35; *p* = 0.003). In the same study, higher relative humidity was associated with lower odds of high viral exposure (OR 0.60, 95% CI 0.46–0.79), underscoring the importance of combining filtration with broader environmental controls [49].

### 8.2. Humidity, Temperature and Material Reservoirs

A growing consensus identifies a relative humidity range of approximately 40 to 60% as protective for respiratory health: viral persistence is reduced, mucociliary clearance is optimised and dust-mite proliferation is restrained [65,131]. Very low humidity below 30% can prolong viral viability and dry respiratory mucosa; very high humidity above 60% favours dust mites and fungal proliferation [65,131]. Temperature interacts with humidity to determine droplet evaporation rates and viral persistence. Most respiratory viruses persist better at lower temperatures, contributing to seasonal patterns of illness [65]. Porous materials, textile-rich furnishings and damp building envelopes harbour and re-emit microorganisms, while metal and copper-alloy surfaces have intrinsic antimicrobial properties [132]. Water systems, including cooling towers, plumbing, condensate trays and humidifiers, are critical control points for Legionella and atypical mycobacteria [82].

### 8.3. Occupancy and Behavioural Determinants

Occupant density, activity level, mask use and behavioural factors interact with engineering controls to determine net exposure. A well-ventilated space can be rendered hazardous by overcrowding or aerosol-generating activities, while a modestly ventilated space can be made safer by reduced occupancy and well-fitted respiratory protection [79,133].

## 9. Prevention, Control and Surveillance Strategies

A coherent strategy for managing the public-health risks of indoor airborne pathogens combines engineering controls, administrative controls, personal protective measures and surveillance, following the hierarchy of controls familiar from occupational health [134].

### 9.1. Engineering and Administrative Controls

Engineering controls constitute the most reliable and least behaviour-dependent layer. Adequate ventilation, high-efficiency filtration of recirculated air, upper-room ultraviolet germicidal irradiation for high-risk settings, humidity control within the 40 to 60% range and demand-controlled ventilation linked to CO_2_ sensors collectively form the engineering toolkit [23,123,128,135]. UVGI has evidence in healthcare and congregate settings for reducing transmission of airborne pathogens and is increasingly considered for schools and other high-density environments [136]. Occupancy management, scheduling of high-aerosol activities, isolation of infectious individuals, telework policies during outbreaks and clear ventilation maintenance protocols all fall into this category [23]. The most consequential administrative interventions during the COVID-19 pandemic, including staying home when symptomatic, isolating cases and reducing density, interacted with engineering controls in determining net risk.

### 9.2. Personal Protection and Vaccination

Well-fitted respirators provide substantial reduction in inhaled aerosol exposure for both healthcare workers and high-risk community members [137]. Surgical and cloth masks reduce emission of aerosols and offer partial filtration of inhaled aerosols. Vaccination, while not strictly an indoor-air intervention, dramatically modifies the consequences of indoor exposure for several pathogens.

### 9.3. Environmental and Pathogen Surveillance

Real-time monitoring of indoor environments is rapidly developing. Carbon dioxide measurement as a proxy for ventilation adequacy is inexpensive and increasingly deployed in schools and workplaces [138]. Particle counters provide more direct, although not pathogen-specific, indicators of aerosol load [139]. Environmental sampling of air, surfaces and wastewater, combined with quantitative PCR or sequencing, offers pathogen-specific surveillance and has been demonstrated for SARS-CoV-2, influenza, RSV and tuberculosis [140,141]. Recent citywide evidence further supports the feasibility of indoor air surveillance. In Chicago, 10 samplers generated 776 weekly air samples across healthcare and congregate-living settings over two respiratory-virus seasons. At least one of four monitored viruses, influenza A, influenza B, RSV or SARS-CoV-2, was detected in 83% of samples, with a mean of 1.7 viruses per sample. Aggregated airborne viral signals closely followed clinical and wastewater surveillance trends, while all 29 SARS-CoV-2 variants tracked through citywide clinical and wastewater surveillance were also identified in air samples from the 10 indoor locations [50]. Wastewater-based epidemiology has emerged as a sensitive complement to clinical surveillance [142].

### 9.4. Policy, Standards and Implementation

WHO, US EPA, ASHRAE, ECDC and national agencies have updated or are updating indoor air guidance in light of pandemic experience [23,80,126]. Building codes, school construction standards and healthcare facility standards are gradually adapting, although implementation lags policy, and investment remains modest relative to the demonstrated burden of disease [143]. A growing argument from public health researchers and engineers calls for indoor air quality to be regulated with the same seriousness as drinking water, with mandatory standards and verification [144].

### 9.5. Emerging Technologies and Integrated Monitoring

The technological landscape for indoor air management is moving quickly. Several developments deserve attention. Laser-induced fluorescence and Raman spectroscopy enable real-time detection of biological particles in air, distinguishing them from inert dust [139,145]. Microfluidic platforms coupled with isothermal nucleic acid amplification may allow pathogen-specific detection at the room scale [146]. These technologies are not yet routine, but their cost is falling, and their resolution is improving. Environmental DNA and RNA sequencing of indoor air filters and dust offers a comprehensive view of microbial communities, allergens and resistance genes [21,28]. Integrated with clinical and wastewater data, such surveillance could inform outbreak detection, ventilation interventions and resistance monitoring at building, neighbourhood and city scales. Building management systems are increasingly capable of integrating CO_2_, particulate matter, volatile organic compound and occupancy data into adaptive control of ventilation, filtration and UVGI [147]. Digital twins of buildings, namely high-fidelity computational replicas, allow simulation of pathogen dispersion under varying scenarios and can support design and operational decisions [148]. Copper-alloy surfaces, photocatalytic coatings, antimicrobial polymers and engineered air-filtration media can complement traditional engineering controls [149,150]. Their introduction must be balanced against AMR and ecological concerns; widespread biocidal exposure can select for resistance and disrupt protective microbial communities [21]. A flourishing market of ionisation, hydroxyl-radical, plasma and photocatalytic devices has emerged, often with inadequate independent evaluation [151]. Regulators, building owners and the public would benefit from rigorous performance and safety standards, including assessment of by-products such as ozone and formaldehyde. The opportunity is to build integrated indoor environmental management systems, combining sensors, filtration, UVGI, ventilation control and microbial surveillance, that can be tuned to the use, occupancy and risk profile of each space.

## 10. Public Health Implications and Future Research Priorities

Several implications for public health follow from the evidence reviewed above. First, indoor air is a major and modifiable determinant of human health. Its biological component is not peripheral to mainstream public health; it is central. The pandemic has shifted public, scientific and political conversation in this direction, but sustained investment is required to consolidate the gains. Second, the framework for indoor air policy is uneven and incomplete. Outdoor air pollution is governed by mandatory standards, regular monitoring and public reporting in much of the world. Indoor air, by contrast, is governed by voluntary guidance, building codes that vary widely and a patchwork of occupational standards focused on a narrow set of agents [143,144]. A more coherent framework, with statutory standards, monitoring and enforcement at least in critical settings such as schools, hospitals, long-term care facilities and workplaces, is warranted. Third, equity considerations must be foundational rather than retrospective. The unequal distribution of indoor environmental burden by income, ethnicity, housing tenure and geography means that any policy response relying solely on private investment in air cleaning, ventilation upgrades or relocation risks widening existing health gaps [111]. Fourth, the research priorities are increasingly clear. They include longitudinal cohorts integrating indoor bioaerosol exposure with immunological, microbiological and clinical biomarkers across the life course; mechanistic studies of trained immunity in human populations with characterised indoor exposures; controlled evaluations of engineering interventions, including UVGI and high-efficiency filtration, on respiratory infection and inflammation outcomes; global studies in low-income and middle-income settings, where much of the disease burden is concentrated but the evidence base remains thin; integration of indoor air, wastewater and clinical surveillance into next-generation pandemic preparedness; and sustained engagement with antimicrobial resistance through indoor environmental measures. Fifth, the disciplinary boundaries that have historically separated building engineering, infectious disease, immunology, environmental health and public health policy must be deliberately bridged.

## 11. Conclusions

Indoor airborne biological exposure extends beyond acute infection and includes repeated contact with viable microorganisms, microbial fragments, allergens and resistance determinants. The strongest evidence supports three conclusions: respiratory viruses are efficiently transmitted through inhalation in indoor spaces; inadequate ventilation, high occupancy and unfavourable environmental conditions increase exposure, while filtration and ventilation reduce airborne particle concentrations; and residential dampness and mould are consistently associated with respiratory symptoms and poorer asthma outcomes. Quantitative field studies identified bacterial and fungal concentrations ranging from hundreds to several thousand CFU/m^3^. However, the effectiveness of individual interventions remains context dependent. For example, HEPA filtration in classrooms reduced viral diversity by 32.8% but did not significantly reduce the odds of high overall viral exposure, supporting the use of layered rather than isolated controls. Moderate mechanistic and epidemiological evidence indicates that endotoxin, β-(1→3)-glucans, fungal proteases and other microbial components can disrupt epithelial barriers and activate innate immune pathways. Nevertheless, direct causal evidence linking chronic low-dose indoor exposure to airway remodelling, systemic inflammation or long-term immune dysregulation remains limited. Similarly, the detection of antimicrobial resistance genes and mobile genetic elements in indoor air confirms that buildings can act as environmental reservoirs, but the contribution of airborne transmission and horizontal gene transfer to clinically relevant AMR spread has not been adequately quantified. Evidence concerning trained immunity, systemic cardiometabolic effects and the long-term consequences of reduced indoor microbial diversity should therefore be considered emerging and hypothesis-generating. This review has several limitations. As a narrative review, it did not apply a formal risk-of-bias assessment or quantitative meta-analysis. Comparisons were restricted by substantial heterogeneity in bioaerosol sampling, particle-size classification, viability assessment, exposure metrics and clinical outcomes. Many studies detected microbial nucleic acids or non-viable material and therefore could not establish infectivity or transmission risk. The evidence base was also concentrated in high-income countries and healthcare, educational or occupational settings, limiting generalisability to low-resource environments and residential populations. Priority research needs include harmonised bioaerosol sampling and reporting protocols, longitudinal cohorts linking repeated indoor measurements with immunological and clinical outcomes, controlled evaluations of combined ventilation and filtration strategies, and direct investigation of airborne AMR transmission and horizontal gene transfer. Greater attention is also required for children, older adults, immunocompromised populations and communities affected by poor housing. Until these gaps are addressed, indoor-air policies should prioritise layered, proportionate interventions combining ventilation, filtration, moisture control, occupancy management and pathogen-specific surveillance.

## Figures and Tables

**Table 1:** Representative indoor airborne biological agents, exposure characteristics, immune pathways and health outcomes.

Biological Agent	Principal Indoor Source	Representative Setting	Relevant Physicochemical Properties	Principal Immune Pathway(s)	Potential Clinical Outcome(s)	Selected Quantitative Findings
**Respiratory viruses (influenza viruses, RSV, rhinoviruses and SARS-CoV-2)**	Infected occupants; breathing, speaking, coughing, singing and respiratory procedures.	Homes; schools; healthcare facilities; care homes; public transport; crowded spaces.	Virions carried in droplets and fine aerosols (<5 μm); persistence modified by relative humidity, temperature and aerosol composition.	TLR3/7/8 and RIG-I/MDA5 → NF-κB and type I/III interferons; epithelial cytokines and neutrophil recruitment.	Acute respiratory infection; asthma and COPD exacerbation; severe disease in vulnerable hosts.	Detected in 98.5% of 532 classroom samples; HEPA reduced viral diversity by 32.8% but not high viral exposure [49]. At least one virus occurred in 644/776 indoor-air samples (83%) [50].
**Viable airborne bacteria (e.g., *Staphylococcus*, *Streptococcus*, *Pseudomonas*, *Legionella* and *Mycobacterium* spp.)**	Occupants; settled dust; surfaces; HVAC systems; aerosolised water reservoirs.	Offices; classrooms; day-care centres; hospitals; crowded housing; water-system-associated environments.	Whole cells or aggregates in coarse and fine particle fractions; deposition depends on aerodynamic diameter; viability is influenced by desiccation and environmental conditions.	TLR2/TLR4 and NOD1/NOD2 → NF-κB/MAPK; IL-1β, IL-6, IL-8 and TNF; neutrophils and NETs.	Acute and healthcare-associated infection; tuberculosis; Legionnaires’ disease; airway inflammation and exacerbations.	924–2750 CFU/m^3^ in university microenvironments [33]; maximum 1668 CFU/m^3^ in day-care centres [44]; 118–259 CFU/m^3^ in hospitals, with a predominant 4.7–5.8 μm fraction [51].
**Fungal spores and hyphal fragments (e.g., *Aspergillus*, *Penicillium*, *Cladosporium*, *Alternaria* and *Stachybotrys* spp.)**	Damp materials; visible mould; settled dust; outdoor-air infiltration; contaminated HVAC components.	Damp homes; schools; day-care centres; offices; hospitals; construction and occupational settings.	Micrometre-sized spores and smaller hyphal fragments; viable and non-viable particles may carry β-glucans, proteases, allergens and mycotoxins.	Dectin-1–Syk–CARD9–NF-κB and TLR signalling; cytokines, neutrophils and Th17 responses; protease-mediated epithelial barrier disruption.	Allergic rhinitis; asthma; hypersensitivity pneumonitis; chronic airway inflammation; invasive aspergillosis in immunocompromised hosts.	656–1799 CFU/m^3^ [33]; maximum 1706 CFU/m^3^ and correlation with relative humidity (ρ = 0.71, *p* < 0.01) [44]. Each log-unit spore increase was associated with TNF-α +6.5% and IL-8 +5% [52].
**Endotoxin and other bacterial cell-wall components**	Gram-negative bacteria in dust, damp materials, agricultural material, occupied spaces and HVAC reservoirs.	Farm homes; damp buildings; schools; offices; occupational environments.	Non-viable, relatively heat-stable components that remain biologically active and can be repeatedly resuspended with dust.	TLR4–CD14–MD-2 through MyD88/TRIF → NF-κB/MAPK; cytokines, neutrophilic inflammation and context-dependent trained immunity or tolerance.	Acute or chronic airway inflammation; asthma and COPD exacerbation; potentially protective early-life immune programming in specific contexts.	Median house-dust endotoxin was 6.8-fold higher in Amish than Hutterite homes, while asthma prevalence was 5.2% versus 21.3%; mixed exposure prevents single-agent attribution [53].
**β-(1→3)-D-glucans**	Fungal cell walls, spores and hyphal fragments in mould-contaminated dust and materials.	Damp homes; schools; offices; agricultural and wood-processing environments.	Non-viable polysaccharides; activity depends on molecular structure, branching, solubility and particle association; may persist after fungal death.	Dectin-1–Syk–CARD9–BCL10–MALT1–NF-κB; IL-1β, IL-6, TNF and IL-17-associated responses; neutrophils and Th17 differentiation.	Airway inflammation; respiratory symptoms; asthma exacerbation; context-dependent inflammatory adaptation.	Pulmonary models demonstrate Dectin-1- and CARD9-dependent macrophage and cytokine responses [54,55]; direct human indoor dose–response data and harmonised thresholds remain unavailable [54,55,56,57].
**Dampness-related fungal allergens, proteases and mycotoxins**	Mould growth; house-dust mites; pets; cockroaches; contaminated settled dust.	Damp homes; schools; workplaces; water-damaged buildings.	Allergenic and proteolytic proteins carried on spores or dust; proteases can disrupt tight junctions; mycotoxins are low-molecular-weight secondary metabolites.	Epithelial barrier disruption and Th2/IgE-mediated responses; oxidative, ribotoxic and inflammatory stress from some mycotoxins.	Allergic sensitisation; rhinitis; asthma exacerbation; hypersensitivity responses; possible toxic or constitutional symptoms.	Among 424 children with recurrent wheeze or asthma, 34% reported home moisture or mould exposure; uncontrolled asthma occurred in 45% of exposed versus 33% of unexposed children (*p* = 0.03) [58].
**Extracellular DNA (eDNA) and microbial extracellular vesicles**	Lysis or active secretion by bacteria and fungi; biofilms; dust; water reservoirs; HVAC systems.	Homes; hospitals; damp buildings; biofilm-containing water and ventilation systems.	Cell-free DNA and nanoscale membrane vesicles are non-viable but may persist in dust or aerosols; vesicles transport microbial proteins, lipids and nucleic acids.	Nucleic-acid-sensing PRRs and inflammasome signalling; NF-κB and cytokines; eDNA may amplify NET-associated inflammation.	Epithelial activation; mucus production; tissue injury; persistent airway inflammation.	Standardised airborne thresholds are unavailable. Increased extracellular DNA and NET burden has been demonstrated in severe asthma, but the fraction attributable to indoor exposure remains unquantified [59].
**Antimicrobial resistance genes (ARGs) and mobile genetic elements (MGEs)**	Resistant bacteria and eDNA from occupants, surfaces, dust, water systems and biofilms.	Hospitals and ICUs; homes; schools; offices; other occupied buildings.	Chromosomal or plasmid-, integron- and transposon-associated DNA; dissemination through conjugation, transformation or transduction; detection does not establish viability or successful transfer.	No specific host immune pathway; the principal mechanism is ecological and genetic, with indirect immune activation when ARGs are carried by viable pathogens.	Persistence and dissemination of AMR; difficult-to-treat infection and potential treatment failure.	Hospital air contained 0.55–479.44 ARG/MGE copies/m^3^, peaking in respiratory wards [51]. ARGs occurred in only 82/38,861 analysed phage genomes [60].

Abbreviations: ARG, antimicrobial resistance gene; CARD9, caspase recruitment domain-containing protein 9; CFU, colony-forming unit; COPD, chronic obstructive pulmonary disease; HEPA, high-efficiency particulate air; HVAC, heating, ventilation and air-conditioning; MAPK, mitogen-activated protein kinase; MGE, mobile genetic element; NET, neutrophil extracellular trap; NF-κB, nuclear factor kappa B; PRR, pattern-recognition receptor; RSV, respiratory syncytial virus; Syk, spleen tyrosine kinase; TLR, Toll-like receptor. Quantitative findings should not be directly compared across studies because sampling, culture, molecular detection and exposure-assessment methods differ. Detection of microbial nucleic acids does not necessarily demonstrate viability, infectivity or causality.

## Data Availability

No new data were created or analysed in this study. Data sharing is not applicable to this article.

## Abbreviations

The following abbreviations are used in this manuscript:

AMR	Antimicrobial resistance
ASHRAE	American Society of Heating, Refrigerating and Air-Conditioning Engineers
CO_2_	Carbon dioxide
COPD	Chronic obstructive pulmonary disease
COVID-19	Coronavirus disease 2019
DNA	Deoxyribonucleic acid
ECDC	European Centre for Disease Prevention and Control
HEPA	High-efficiency particulate air
HVAC	Heating, ventilation and air conditioning
LPS	Lipopolysaccharide
MAPK	Mitogen-activated protein kinase
MERS-CoV	Middle East respiratory syndrome coronavirus
MERV	Minimum efficiency reporting value
NF-κB	Nuclear factor kappa-light-chain-enhancer of activated B cells
PCR	Polymerase chain reaction
RNA	Ribonucleic acid
RSV	Respiratory syncytial virus
SARS-CoV-1	Severe acute respiratory syndrome coronavirus 1
SARS-CoV-2	Severe acute respiratory syndrome coronavirus 2
US EPA	United States Environmental Protection Agency
UVGI	Ultraviolet germicidal irradiation
WHO	World Health Organization
